# Mitochondrial HSC70-1 Regulates Polar Auxin Transport through ROS Homeostasis in Arabidopsis Roots

**DOI:** 10.3390/antiox11102035

**Published:** 2022-10-15

**Authors:** Tingting Shen, Ning Jia, Shanshan Wei, Wenyan Xu, Tingting Lv, Jiaoteng Bai, Bing Li

**Affiliations:** 1 Ministry of Education Key Laboratory of Molecular and Cellular Biology, Hebei Collaboration Innovation Center for Cell Signaling and Environmental Adaptation, Hebei Key Laboratory of Molecular and Cellular Biology, College of Life Sciences, Hebei Normal University, Shijiazhuang 050024, China; 2 National Key Laboratory of Plant Molecular Genetics, CAS Center for Excellence in Molecular Plant Sciences, Shanghai Institute of Plant Physiology and Ecology, Chinese Academy of Sciences, Shanghai 200032, China; 3 College of Life Sciences, Hengshui University, Hengshui 053000, China

**Keywords:** mtHSC70-1, polar auxin transport, reactive oxygen species, PIN, AUX1, *Arabidopsis thaliana*

## Abstract

Arabidopsis mitochondrial-localized heat shock protein 70-1 (mtHSC70-1) modulates vegetative growth by assisting mitochondrial complex IV assembly and maintaining reactive oxygen species (ROS) homeostasis. In addition, mtHSC70-1 affects embryo development, and this effect is mediated by auxin. However, whether mtHSC70-1 regulates vegetative growth through auxin and knowledge of the link between ROS homeostasis and auxin distribution remain unclear. Here, we found that *mtHSC70-1* knockout seedlings (*mthsc70-1a*) displayed shortened roots, decreased fresh root weight and lateral root number, increased root width and abnormal root morphology. The introduction of the *mtHSC70-1* gene into *mthsc70-1a* restored the growth and development of roots to the level of the wild type. However, sugar and auxin supplementation could not help the mutant roots restore to normal. Moreover, *mthsc70-1a* seedlings showed a decrease in meristem length and activity, auxin transport carrier (PINs and AUX1) and auxin abundances in root tips. The application of exogenous reducing agents upregulated the levels of PINs in the mutant roots. The introduction of antioxidant enzyme genes (*MSD1* or *CAT1*) into the *mthsc70-1a* mutant rescued the PIN and local auxin abundances and root growth and development. Taken together, our data suggest that mtHSC70-1 regulates polar auxin transport through ROS homeostasis in *Arabidopsis* roots.

## 1. Introduction

Reactive oxygen species (ROS), including superoxide anions (O_2_^−^) and hydrogen peroxide (H_2_O_2_), have received increasing attention as novel signal molecules that are involved in growth, differentiation, cell death and response to environmental stresses [1,2,3]. At present, it is clearly realized that there must be coordinated functioning of the signaling networks that govern ROS responses [4]. Under a physiological state, ROS levels are in dynamic balance, and ROS homeostasis is achieved through its production and scavenging [3,5,6]. Plants possess many ROS-scavenging enzymes, such as ascorbate peroxidases (APXs), catalases (CATs), peroxidases (POXs), superoxide dismutases (SODs) and some small molecular antioxidants, such as ascorbate (ASC, vitamin C), tocopherol (vitamin E), beta-carotene and glutathione (GSH) [6]. However, persistently high ROS levels that exceed the antioxidant capacities of cells are toxic and can cause oxidative damage [5,7]. It is well established that supraphysiological concentrations of ROS react non-specifically with proteins, lipids, nucleic acids and carbohydrates and generate other reactive species with potentially toxic consequences [8,9]. Moreover, high levels of ROS inhibit cell elongation, likely by stiffening cell walls [10]. Another possibility is that high levels of ROS inhibit plant growth and development by downregulating the expression levels of auxin-related genes [11,12,13,14,15,16,17]. Analysis of auxin response gene expression indicates that the levels of several auxin receptors and Aux/IAA transcriptional repressors decrease with the increase of extracellular ROS [14].

The phytohormone auxin (indole-3-acetic acid, IAA) is considered a general coordinator of growth and development and is used throughout the life cycle of plants to mediate communication between cells and tissues [18]. Auxin is distributed in plant bodies by two different but interrelated transport systems: (1) a rapid, undirected stream in the phloem and (2) slow and directed intercellular polar auxin transport (PAT) [19]. The PAT system distributes auxin in a very accurate manner and is mediated by auxin influx and efflux carriers. AUXIN1/LIKE-AUX1 (AUX1/LAX) family members are capital auxin influx carriers. PINFORMED (PIN) family members are principal auxin efflux carriers [20,21]. There are eight PIN genes in the *Arabidopsis thaliana* genome, which can be divided into two subfamilies: ‘long’ PINs and ‘short’ PINs. The long PINs include PIN1-PIN4, PIN6 and PIN7, which are localized in the plasma membranes. The short PINs include AtPIN5 and AtPIN8, which are located in the endoplasmic reticulum [22]. The functions of most long PINs have been studied. They are located polarly on different sides of various types of cells [23]. Their polarity determines the direction of intercellular free IAA flow [22]. PAT is necessary for the establishment of embryonic apical-basal polarity, organogenesis, organ positioning, root patterning and hypocotyl elongation [24,25,26]. *PIN* mutants display defective developmental phenotypes. For example, *pin1* mutants can have fused cotyledons, three cotyledons, pin-like inflorescence and abnormal flowers [27], *pin2* mutants show non-gravitropic root growth [28], triple-mutant *pin1*,*3*,*4* seedlings display fused, cup-shaped cotyledons and multiple mutant *pin1*,*3*,*4*,*7* embryos display a no apical-basal patterning [29,30]. Carrier-mediated PAT can be regulated at multiple levels, such as mRNA transcription, subcellular distribution, transport activity and protein degradation [22].

All organisms respond to high temperature stress by producing heat-shock proteins (HSPs). HSP70s are the most abundant type of HSP, which form pivotal links in the molecular chaperone network that regulates every aspect of cellular protein homeostasis, such as nascent protein folding, protein translocation and assembly, refolding of stress-denatured proteins and degradation of toxic protein aggregates [31,32,33]. *A. thaliana* contains 14 HSP70 genes, which are highly conserved and localized in distinct subcellular compartments, including the cytosol, mitochondria, plastids, endoplasmic reticulum (ER) and nucleus [34,35,36]. They participate in regulating numerous biological processes as molecular chaperons [37,38,39,40,41,42,43,44]. The molecular and physiological functions of mitochondrial HSP70s (mtHSC70s) have been reported in yeast, animals and plants. In plants, the overexpression of *mtHSC70* inhibits heat stress and H_2_O_2_ stress-induced programmed cell death in *Oryza sativa* protoplasts [45]. *A. thaliana* mtHSC70-1 has weak ATPase activity, which is stimulated by mitochondrial J-domain protein AtDjB1 [46]. Wei et al. [47] and Zhai et al. [48] reported that *A. thaliana mtHSC70-1* knockout leads to severe growth inhibition and an increase in mitochondrial ROS levels during the vegetative growth of plants and that mtHSC70-1 is directly involved in respiratory chain complex IV/cytochrome c oxidase (COX) assembly and the establishment of COX-dependent respiration. Li et al. [49] reported that *A. thaliana mtHSC70-1* knockout impairs embryo development, and this effect is mediated by auxin. Moreover, the loss of function of mtHSC70-1 induces mitochondrial retrograde signaling, which reduces the expression levels of auxin biosynthesis and PAT genes, resulting in abnormal auxin gradients in the embryo and defective embryo development [49]. However, the role of mtHSC70-1 in regulating PAT during the vegetative growth of plants remains unclear. Here, we investigated the effects of *mtHSC70-1* knockout on PAT in *A. thaliana* roots and the role of ROS homeostasis in the maintenance of PAT.

## 2. Materials and Methods

### 2.1. Plant Materials and Growth Conditions

*Arabidopsis thaliana* (ecotype Columbia-0) seeds were sterilized with 75% ethanol and sown in 0.5 × Murashige–Skoog (MS) medium [50] including 1.0% (*w*/*v*) sucrose and 0.8% (*w*/*v*) agar. The sterilized seeds were kept at 4 °C for 3 d and then cultured in a growth chamber at 22 °C with a 16 h/8 h photoperiod and a light intensity of approximately 100 μmol m^−2^ s^−1^. After two weeks of growth on MS medium, the seedlings were transplanted into the soil for cultivation in a greenhouse under the same conditions. The cultivated plants were watered once a week.

Col*/PIN:PIN-GFP* (the *PIN* represents *PIN1*, *PIN2*, *PIN3* or *PIN7*), Col*/AUX1:AUX1-YFP*, Col*/DR5:GFP* and Col*/DR5:GUS* seeds were gifts from Prof. Zhenbiao Yang. The seeds of two putative T-DNA insertional mutant lines for *mtHSC70-1* (At4g37910), *mthsc70-1a* (SALK_081383) and *mthsc70-1b* (SALK_081385) were obtained from the Arabidopsis Biological Resource Center (ABRC, Columbus, OH, USA). The homozygous *mthsc70-1a* and *mthsc70-1b* mutants and transgenic plants *mthsc70-1a/PIN:PIN-GFP* (the *PIN* represents *PIN1*, *PIN2*, *PIN3* or *PIN7*), *mthsc70-1a/AUX1:AUX1-YFP*, *mthsc70-1a*/*DR5:GFP*, *mthsc70-1a/DR5:GUS*, R2/3 (two *mthsc70-1a*/*mtHSC70-1*:*mtHSC70-1* lines), MSD1-R2/3 (two *mthsc70-1a*/*mtHSC70-1*:*MSD1* lines) and CAT1-R2/3 (two *mthsc70-1a*/*mtHSC70-1*:*CAT1* lines) were generated in our previous study [47].

### 2.2. Hybridization of Plants with Different Genotypes

The emasculated flowers from Col plants harboring *PIN:PIN-GFP* (the *PIN* represents *PIN1*, *PIN2*, *PIN3* or *PIN7*), *AUX1:AUX1-YFP* or *DR5:GFP* construct were crossed with pollen from *mthsc70-1a* plants, which generated *mthsc70-1a*/*PIN1-GFP*, *mthsc70-1a*/*PIN2-GFP*, *mthsc70-1a*/*PIN3-GFP*, *mthsc70-1a*/*PIN7-GFP*, *mthsc70-1a*/*AUX1-YFP* and *mthsc70-1a*/*DR5:GFP* plants. Identification of the homozygous *mthsc70-1a* background was performed by PCR using insertional site-specific primers for *mthsc70-1a* and a T-DNA left border primer, LBb1.3 (Table S1). Identification of the GFP or YFP background was performed by observing the GFP or YFP fluorescence; those lines in which all seedlings showed fluorescence were used for the experiments. GFP or YFP fluorescence was observed using a fluorescence microscope (ECLIPSE 80i, Nikon, Japan).

To generate *mthsc70-1a*/*PIN:PIN-GFP* (the *PIN* represents *PIN1*, *PIN2*, *PIN3* or *PIN7*) or *mthsc70-1a*/*DR5:GFP* plants harboring the *mtHSC70-1*, *MSD1* or *CAT1* gene, the emasculated flowers from R2 plants (a *mthsc70-1a/mtHSC70-1:mtHSC70-1* line), MSD1-R2 plants (a *mthsc70-1a/mtHSC70-1:MSD1* line) or CAT1-R2 plants (a *mthsc70-1a/mtHSC70-1:CAT1* line) were crossed with pollen from *mthsc70-1a*/*PIN:PIN-GFP* plants or *mthsc70-1a*/*DR5:GFP* plants. Identification of the homozygous *mtHSC70-1*, *MSD1* or *CAT1* background was performed by a hygromycin resistance screen. Identification of the *PIN-GFP* (or *DR5:GFP*) background was performed by observing the GFP fluorescence; those lines in which all seedlings showed fluorescence were used for the experiments. GFP fluorescence was observed using a fluorescence microscope (ECLIPSE 80i, Nikon, Japan).

Genotypes of the transgenic lines used in this study are indicated in Table S2.

### 2.3. Methods of EdU Staining, FM4-64 Staining and GUS Staining

Ethynyl-29-deoxyuridine (EdU) staining was conducted as described by Xiong et al. [51] using an EdU detection cocktail (Life Technologies, Carlsbad, CA, USA). In brief, the roots of 4 days old seedlings grown at 22 °C were incubated in 1 μM EdU for 30 min and then fixed in phosphate-buffered saline (PBS, pH 7.2) including 4 % (*w*/*v*) formaldehyde and 0.1% Triton X-100 for 30 min, which was followed by 3 washes with PBS. The fixer was incubated in an EdU detection cocktail for 30 min in the dark and then washed 3 times with PBS.

FM4-64 staining was conducted as described by Gao et al. [52]. In brief, the roots of 4 days old seedlings were incubated in a staining solution containing 5 μM of the plasma membrane marker FM4-64 (Life Technologies, Carlsbad, CA, USA) for 15 min in the dark, which was followed by washing with PBS (pH 7.2).

Analysis for β-glucuronidase (GUS) activity was performed as described by Wei et al. [47]. In brief, seedlings were incubated in GUS histochemical dye liquor [2 mM X-Gluc (Sigma, St Louis, MO, USA) in 50 mM Na_2_HPO_4_/NaH_2_PO_4_ at pH 7.2, 2 mM K_3_Fe(CN)_6_, 2 mM K_4_Fe(CN)_6_, 10 mM EDTA and 0.2% (*v*/*v*) Triton X-100] at 22 °C for 4 h, which was followed by a wash with 70% ethanol.

### 2.4. Observation or Quantification of the Fluorescence Intensity or Distribution in the Stained or Transgenic Roots

The stained or transgenic samples were observed using a laser scanning confocal microscopy (LSCM) (FV3000, Olympus, Tokyo, Japan) with excitation/emission wavelengths of 561/572 nm for FM4-64, 488/510 nm for GFP and EdU and 510/527 nm for YFP, and photographed in their largest z planes. The same parameter settings were used for all genotypic samples in the same experiment. For GUS activity, the stained samples were observed using a stereomicroscope (SZX2-ILLT, Olympus, Tokyo, Japan). Fluorescence in the area of the root shown in the figure was visualized with ImageJ 1.42q software (National Institutes of Health, Bethesda, MD, USA).

### 2.5. Real-Time Quantitative RT–PCR

Ten days old *A. thaliana* seedlings grown at 22 °C and TriPure Reagent (Aidlab Biotechnologies Co., Ltd., Beijing, China) were used for the isolation of total RNA. Real-time quantitative RT–PCR (Q–PCR) was conducted following the method of Zhang et al. [53]. Primer Express software (Applied Biosystems, Carlsbad, CA, USA) was used to design Q-PCR primers (Table S1). PCR was conducted using an ABI Prism 7000 Sequence Detection System (Applied Biosystems, Carlsbad, CA, USA). The expression levels of genes tested in the WT or control plants were set to 1. *ACTIN1* gene was used as the internal control.

### 2.6. Statistical Analysis

STATISTICA 6.0 software (StatSoft, Inc., Tulsa, OK, USA) was used for statistical analyses. Difference significance was tested at *p* < 0.05 by ANOVA with Tukey’s HSD test or Student’s *t*-test.

### 2.7. Accession Numbers

*mtHSC70*-*1*, At4G37910; *MSD1*, At3G10920; *CAT1*, At1G20630; *PIN1*, At1G73590; *PIN2*, At5G57090; *PIN3*, At1G70940; *PIN7*, At1G23080; *AUX1*, At2G38120; *mthsc70-1a*, SALK_081383; *mthsc70-1b*, SALK_081385.

## 3. Results

### 3.1. Role of Glucose in Improving the Growth and Development of mtHSC70-1 Mutant Roots

**Figure 1:**
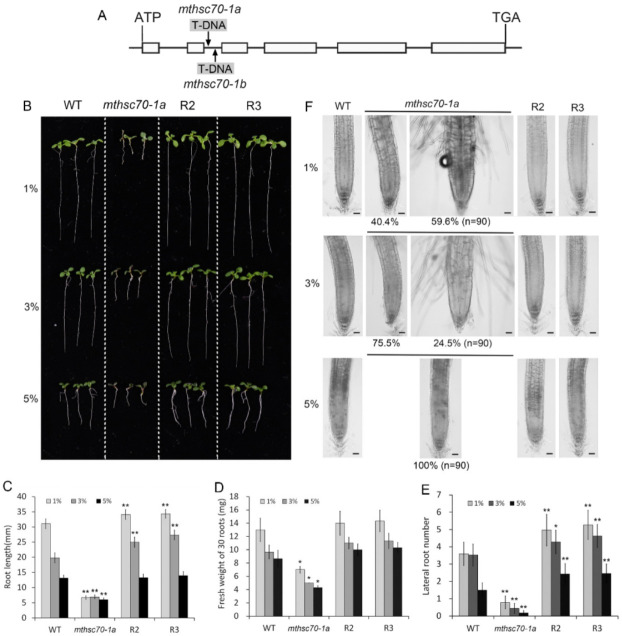
The effects of glucose on root growth and development in wild type, *mthsc70-1a*, and two complementary *mthsc70-1a* lines. (**A**) Intron/exon organization of the *mtHSC70-1* CDS and T-DNA insertion locations. Boxes, exons; lines, introns; arrows, T-DNA insertion positions. (**B**) Representative seedlings after 10 days of growth in 0.5 × MS medium supplemented with 1%, 3% or 5% glucose. (**C**–**E**) Comparison of root length (**C**), fresh weight (**D**) and lateral root number (**E**) among WT, *mthsc70-1a*, R2 and R3 seedlings. The asterisks indicate significant differences from WT of the same treatment (*t*-test, *, *p <* 0.05; **, *p <* 0.01). (**F**) The roots of 10 days old seedlings grown in 0.5 × MS medium supplemented with 1%, 3% or 5% glucose were observed using a laser scanning confocal microscopy (LSCM). Representative images are shown. Scale bar = 40 µm. The roots of the *mtHSC70-1* mutants were divided into two types according to their width (≤140 µm or >140 µm) in the transition position of the meristem zone and elongation zone. The percentages below the images represent the ratio of two types of roots. n, the total number of roots analyzed. WT, wild type; *mthsc70-1a/b*, two *mtHSC70-1* T-DNA insertion mutant lines; R2 and R3, two complementary *mthsc70-1a* lines (*mthsc70-1a*/*mtHSC70-1*:*mtHSC70-1*).

*A. thaliana mtHSC70-1* mutants exhibit higher total respiration, AOX respiratory pathway and ATP level, lower COX-dependent respiratory pathway and shorter roots compared to wild-type plants [47]. Glucose is the original substrate of the glycolysis pathway related to mitochondrial respiration and energy metabolism, so it can promote respiration and energy production. To confirm that the defects of *mtHSC70-1* mutant roots are not due to energy deficiency, we needed to know whether sugar supplementation may rescue the growth and development of the mutant roots. To clarify this topic, we compared the phenotypes of roots of WT, *mtHSC70-1* mutant (*mthsc70-1a*, Figure 1A) and two complementary *mthsc70-1a* lines (R2 and R3) grown on 0.5 × MS medium supplemented with different concentrations of glucose. In MS medium containing 1% glucose, the primary root of *mthsc70-1a* was 78.5% shorter than that of wild-type seedlings, with a 46% reduction in fresh weight and fewer lateral roots (Figure 1B–E). Moreover, 59.6% of *mthsc70-1a* roots showed abnormal morphology, such as longer root hairs near the root tips, increased root width (>140 µm), shorter meristem length and larger and disordered cell arrangement in the elongation zone (Figure 1F; Figure S1). Two complementary *mthsc70-1a* lines showed phenotypes similar to those of wild-type roots (Figure 1B–F), indicating the roles of mtHSC70-1 in regulating root growth and development. Compared with 1% glucose, 3% and 5% glucose reduced primary root length, fresh root weight and lateral root number to some extent in all analyzed genotypic seedlings; however, they did not recover the root length, fresh weight and lateral root number of *mthsc70-1a* to WT level (Figure 1B–E). In contrast, 3% glucose reduced the percentage of roots with abnormal morphology (24.5%); 5% glucose completely inhibited the emergence of roots with abnormal morphology (Figure 1F). These results indicate that sugar supplementation could improve the morphogenesis of the mutant roots but had no positive effect on the root growth of the mutants.

### 3.2. Knockout of the mtHSC70-1 Gene Interferes with Polar Auxin Transport in Roots

**Figure 2:**
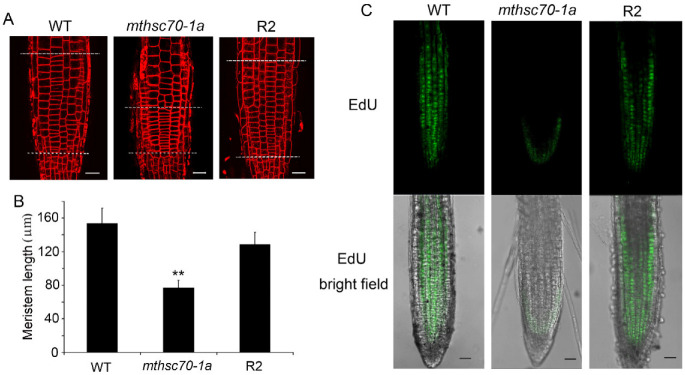
*mtHSC70-1* knockout decreased the meristem length and mitosis activity in root tips. (**A**) The cell files of primary root tips by FM4-64 staining. The roots of 5 days old seedlings were observed using a LSCM. Representative images are shown. The cells without obvious elongation in the stele area were used as the standard of meristem cells in this experiment. The zones between two white dotted lines were measured. (**B**) Meristem lengths of the primary root tips. The data are the means ± SD of 30 roots. The experiments were repeated three times with similar results. The asterisks indicate significant differences from WT (*t*-test, **, *p <* 0.01). (**C**) Results of S-phase entry of primary root tips by EdU staining. The roots of 6 days old seedlings were observed using a LSCM. Representative images are shown. Top row, EdU staining; bottom row, merge of EdU fluorescence and bright field. WT, wild-type; *mthsc70-1a*, a *mtHSC70-1* mutant line; R2, a complementary *mthsc70-1a* line. Scale bar = 20 µm.

**Figure 3:**
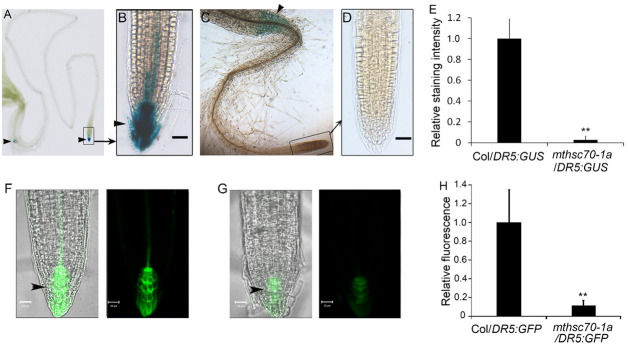
*mtHSC70-1* knockout decreased the auxin response in primary root tips. (**A**–**E**) Detection of GUS activity. Roots of 5 days old Col/*DR5:GUS* (**A**,**B**) or *mthsc70-1a/DR5:GUS* (**C**,**D**) seedlings were incubated in GUS dye liquor and observed using a stereomicroscope. Representative images are shown. Figure (**B**,**D**) are enlarged views of the rectangular frames in Figure (**A**,**C**), respectively. Scale bar = 40 µm. The staining intensities in root tips were visualized with ImageJ 1.42q software (National Institutes of Health, Bethesda, MD, USA) (**E**). Thirty seedlings from each genotype were analyzed. The asterisk indicates a significant difference from Col/*DR5:GUS* (*t*-test; **, *p <* 0.01). (**F**–**H**) Detection of GFP fluorescence. Roots of 5 days old Col/*DR5:GFP* (**F**) or *mthsc70-1a/DR5:GFP* (**G**) seedlings were observed using a LSCM. Representative images are shown. Scale bar = 20 µm. The fluorescence intensities in root tips were visualized with ImageJ 1.42q software (**H**). Thirty seedlings from each genotype were analyzed. The asterisk indicates significant difference from Col/*DR5:GFP* (*t*-test; **, *p <* 0.01). Arrowheads point to the locations of the GUS or GFP. Col/*DR5:GUS*, Col plants harboring *DR5:GUS*; *mthsc70-1a/DR5:GUS*, *mthsc70-1a* plants harboring *DR5:GUS*; Col/*DR5:GFP*, Col plants harboring *DR5:GFP*; *mthsc70-1a/DR5:GFP*, *mthsc70-1a* plants harboring *DR5:GFP*.

Auxin is a major regulator of root system architecture and is known to regulate meristematic activities [11,12]. To clarify whether mtHSC70-1 affects root architecture by the auxin pathway, we compared the differences in meristem zone lengths and mitosis activities among WT, *mthsc70-1a* and R2 seedlings. Using FM4-64 (a plasma membrane marker dye) staining to highlight the cell boundaries, we found a pronounced decrease in the length of the meristem zone in the *mthsc70-1a* roots compared to the WT and R2 roots (Figure 2A,B; Figure S1). Using the thymidine analogue EdU for in situ detection of cell cycle S-phase entry, we demonstrated that *mtHSC70-1* knockout greatly reduced root meristem activity (Figure 2C). These results suggest lower cell division rates in the meristems of *mthsc70-1a* roots and imply that the auxin abundances in *mthsc70-1a* root tips might be lower than those of WT and R2 root tips. To confirm this, we crossed the *mthsc70-1a* plants with Col-0 plants harboring *DR5:GFP* or *DR5:GUS* (the synthetic auxin reporter) [24] and obtained homozygous *mthsc70-1a*/*DR5:GFP* plants or *mthsc70-1a*/*DR5:GUS* plants. The intensity of GFP fluorescence or GUS coloration was used to indicate the local auxin abundances in roots. Five days old Col/*DR5:GUS* seedlings exhibited a strong blue coloration in the quiescent center cells, columella cells and stele cells of the root tips (Figure 3A,B). However, in 5 days old *mthsc70-1a*/*DR5:GUS* seedlings, the blue coloration was restricted to the root/hypocotyl transition zone, and no blue coloration was observed in the root tips (Figure 3C–E). Five days old Col/*DR5:GFP* seedlings showed bright GFP fluorescence in the quiescent center cells, columella cells and stele cells (Figure 3F). However, the GFP fluorescence was markedly weaker in the quiescent center cells and columella cells of *mthsc70-1a*/*DR5:GFP* roots (Figure 3G,H). The results indicate that *mtHSC70-1* knockout leads to decreased auxin abundance in root tips.

**Figure 4:**
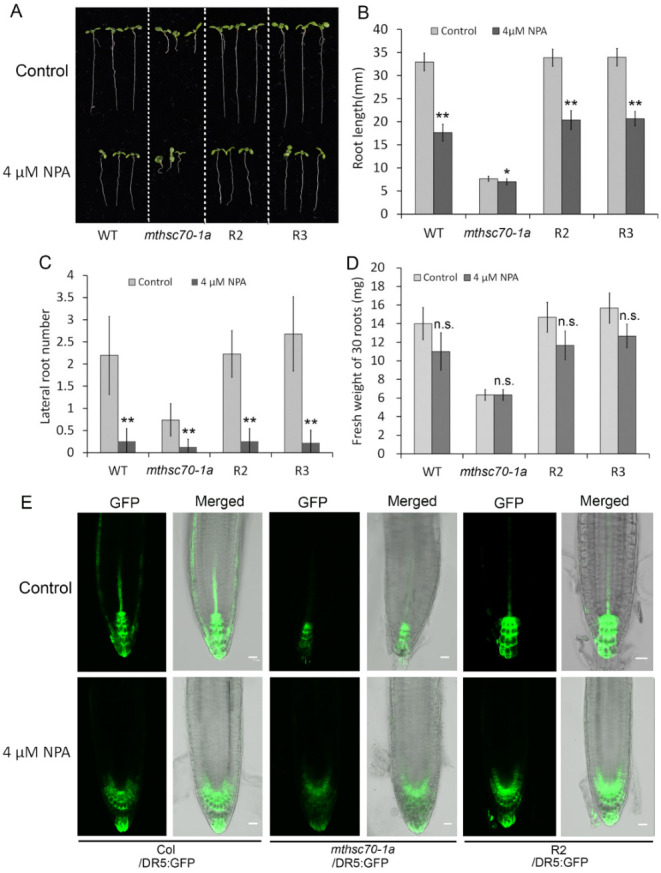
The effects of NPA treatment on root growth and auxin distribution. (**A**–**D**) The effect of NPA treatment on root growth in WT, *mthsc70-1a*, R2 and R3 seedlings. Representative seedlings after 10 days of growth in 0.5 × MS medium with or without 4 µM NPA and shown (**A**). Comparison of root length (**B**), lateral root number (**C**) and fresh weight (**D**) among WT, *mthsc70-1a*, R2 and R3 seedlings. The asterisks indicate significant differences from control group with the same genotype (*t*-test; *, *p <* 0.05; **, *p <* 0.01). n.s., no significant differences from the control group. (**E**) The effect of NPA treatment on auxin distribution in root tips. Roots of 5 days old Col/*DR5:GFP*, *mthsc70-1a/DR5:GFP* and R2/*DR5:GFP* seedlings grown in 0.5 × MS medium with or without 4 µM NPA were observed using a LSCM. Representative images are shown. Scale bar = 20 µm. WT, wild type; *mthsc70-1a*, a *mtHSC70-1* mutant line; R2 and R3, two complementary *mthsc70-1a* lines; Col/*DR5:GFP*, Col plants harboring *DR5:GFP*; *mthsc70-1a/DR5:GFP*, *mthsc70-1a* plants harboring *DR5:GFP*; R2/*DR5:GFP*, R2 plants harboring *DR5:GFP*.

We wanted to know whether the defects of *mtHSC70-1* mutant roots are due to the reduction of auxin synthesis or the blockage of PAT. To clarify this topic, we needed to investigate the effects of auxin supplementation or PAT inhibitor treatment on root growth and auxin distribution. Firstly, we compared the root length of WT and *mthsc70-1a* grown on 0.5 × MS medium, supplemented with different concentrations of 1-naphthlacetic acid (NAA), a synthetic auxin. The results showed that the treatment with exogenous NAA did not rescue the growth of the mutant roots (Figure S2), implying that the root growth defects of *mthsc70-1a* may not be caused by the reduction of auxin synthesis. Subsequently, the effects of auxin transport inhibitor N-1-naphthylphthalamic acid (NPA) on root growth and auxin distribution were investigated. The WT, *mthsc70-1a*, R2 and R3 seeds were germinated and cultured on 0.5 × MS medium supplemented with or without 4 μM NPA. After 10 d of treatment, the root length and lateral root number of WT, R2 and R3 seedlings were significantly decreased compared with those of untreated seedlings, mimicking those of *mthsc70-1a* seedlings (Figure 4A–C), implying that the defects of *mthsc70-1a* in root elongation and lateral root emergence may be due to the blockage of PAT. However, treatment with NPA had no significant effect on fresh root weight in all detected genotypic seedlings (Figure 4D), implying a weaker role of PAT in substance accumulation. Further, the effect of NPA on auxin response was investigated using transgenic plants expressing *DR5:GFP*. After 5 d of NPA treatment, the distribution of GFP fluorescence in root tips was significantly different from those of untreated seedlings for Col/*DR5:GFP*, *mthsc70-1a*/*DR5:GFP* and R2/*DR5:GFP* plants. After NPA treatment, GFP fluorescence was accumulated in the quiescent center zone and reduced markedly in columella cells and stele cells (Figure 4E), indicating that the auxin output from the quiescent center is affected. In addition, we noticed that NPA treatment only caused the change of GFP fluorescence distribution in root tips, and did not obviously reduce the fluorescence intensity. However, the knockout of *mtHSC70-1* not only changed the GFP fluorescence distribution in root tips, but also significantly reduced the fluorescence intensity. The above results suggest that *mtHSC70-1* knockout is likely to interfere with PAT in the roots.

**Figure 5:**
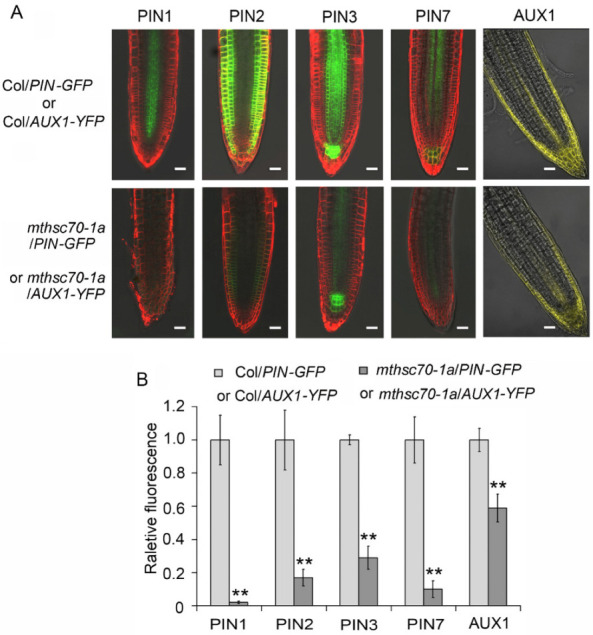
*mtHSC70-1* knockout decreased the abundance of auxin transport carriers in root tips. (**A**) The roots of 5 days old transgenic seedlings harboring *PIN-GFP* were incubated in 5 μM FM4-64 dye. The fluorescence of the transgenic roots harboring *PIN-GFP* or *AUX1-YFP* was observed using a LSCM. Representative images are shown. Scale bar = 20 µm. (**B**) The fluorescence intensities were visualized with ImageJ 1.42q software (National Institutes of Health, Bethesda, MD, USA). The asterisks indicate significant differences from Col/*PIN-GFP* or Col/*AUX1-YFP* (*t*-test; **, *p <* 0.01). Col/*PIN-GFP*, Col/*PIN1-*, *2-*, *3- or 7-GFP* plants; *mthsc70-1a*/*PIN-GFP*, *mthsc70-1a*/*PIN1-*, *2-*, *3- or 7-GFP* plants; Col/*AUX1-YFP*, Col/*AUX1-YFP* plants; *mthsc70-1a*/*AUX1-YFP*, *mthsc70-1a*/*AUX1-YFP* plants.

To confirm the role of mtHSC70-1 in regulating PAT, the abundances of several genes responsible for PAT in root tips were detected, including four PIN genes and an *AUX1* gene. The Col and *mthsc70-1a* plants expressing *PIN1:PIN1-GFP*, *PIN2:PIN2-GFP*, *PIN3:PIN3-GFP*, *PIN7:PIN7-GFP* or *AUX1:AUX1-YFP* (Col/*PIN1-*, *2-*, *3-* or *7-GFP*, Col/*AUX1-YFP*, *mthsc70-1a*/*PIN1-*, *2-*, *3-* or *7-GFP*, *mthsc70-1a*/*AUX1-YFP*) were subjected to visualization of the PIN or AUX1 protein levels (as indicated by GFP or YFP fluorescence). The four *mthsc70-1a*/*PIN-GFP* plants and *mthsc70-1a*/*AUX1-YFP* plants grown at 22 °C for 5 d showed pronounced decreases in fluorescence intensity compared to the corresponding Col/*PIN-GFP* (or Col/*AUX1-YFP*) plants (Figure 5). These results suggest that mtHSC70-1 plays a positive role in regulating the protein levels of PIN1, PIN2, PIN3, PIN7 or AUX1 in root tips and that mtHSC70-1 probably affects root development by regulating PAT.

### 3.3. Role of Exogenous Antioxidants in Restoring Polar Auxin Transport in mtHSC70-1 Mutant Roots

**Figure 6:**
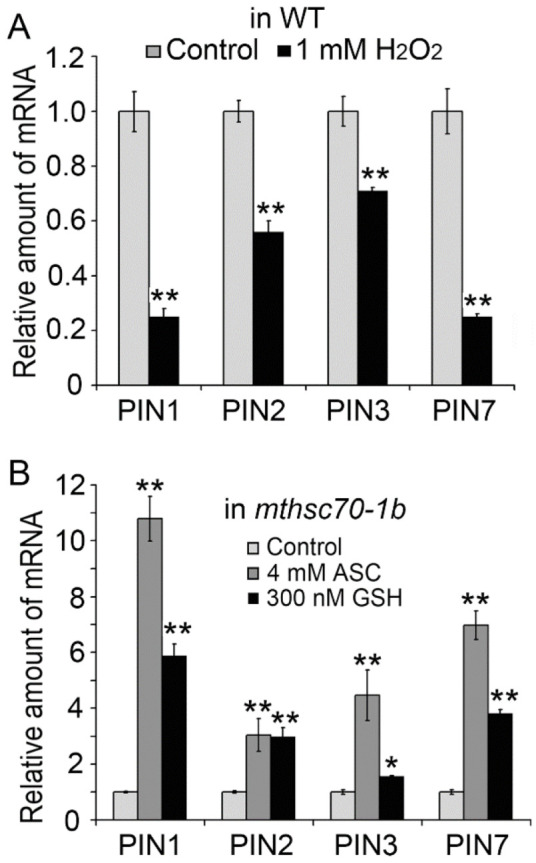
Effects of exogenous redox reagents on the expression of *PIN genes* in WT and *mtHSC70-1* mutant seedlings by Q-PCR. (**A**) The WT seedlings were grown on 0.5 × MS medium with or without 1 mM H_2_O_2_ for 10 d. (**B**) The *mthsc70-1b* seedlings were grown on 0.5 × MS medium with or without 300 nM GSH for 10 d. For the ASC treatment, 10 days old *mthsc70-1b* seedlings were transferred to liquid 0.5 × MS medium with or without 4 mM ASC for 90 min and then developed for 120 min. The data are the means ± SD of three biological replicates. The asterisks indicate significant differences from the control group (*t*-test, *, *p <* 0.05; **, *p <* 0.01). WT, wild-type; *mthsc70-1b*, a *mtHSC70-1* mutant line.

**Figure 7:**
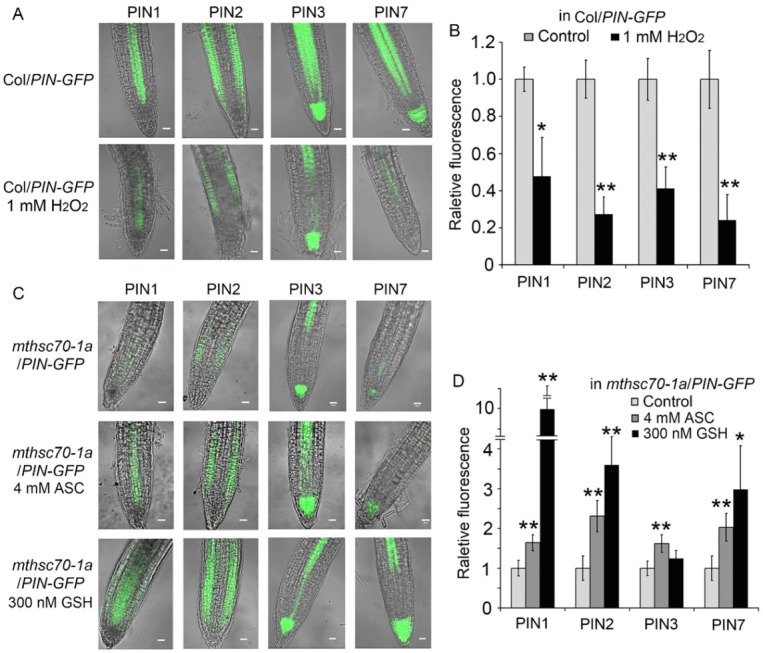
Effects of exogenous redox reagents on PIN protein levels in Col/*PIN-GFP* and *mthsc70-1a*/*PIN-GFP* root tips. (**A**,**B**) Root tips of five days old Col/*PIN-GFP* seedlings grown on 0.5 × MS medium with or without 1 mM H_2_O_2_ were observed using a LSCM (**A**); the GFP fluorescence intensities were visualized with ImageJ 1.42q software (National Institutes of Health, Bethesda, MD, USA) (**B**). (**C**,**D**) Root tips of five days old *mthsc70-1a*/*PIN-GFP* seedlings grown on 0.5 × MS medium with or without 300 nM GSH (or 4 mM ASC) were observed using a LSCM (**C**); the GFP fluorescence intensities were visualized with ImageJ 1.42q software (**D**). Thirty seedlings from each genotype and treatment were analyzed; representative images are shown. Scale bar = 20 µm. The asterisks indicate significant differences from the control group (*t*-test, *, *p <* 0.05; **, *p <* 0.01). Col/*PIN-GFP*, Col/*PIN1-*, *2-*, *3- or 7-GFP* plants; *mthsc70-1a*/*PIN-GFP*, *mthsc70-1a*/*PIN1-*, *2-*, *3- or 7-GFP* plants.

Mitochondria is a major site for redox reactions. To understand the contribution of the cellular redox state on the defected PAT in the *mtHSC70-1* mutant, we investigated the effects of exogenous redox reagents on the PIN and auxin levels in WT and *mtHSC70-1 mutants*. H_2_O_2_ and ASC/GSH were used to increase or decrease the ROS levels in cells. After treatment with 1 mM H_2_O_2_, the WT seedlings displayed a strong downregulation in the expressions of the *PIN1*, *PIN2*, *PIN3* and *PIN7* genes (Figure 6A); however, the *mtHSC70-1* mutant seedlings did not show an obvious difference in *PIN* expression levels from the untreated seedlings (Figure S3A). After treatment with 4 mM ASC or 300 nM GSH, the *mtHSC70-1* mutant seedlings showed a marked upregulation in the expressions of the *PIN1*, *PIN2*, *PIN3* and *PIN7* genes (Figure 6B); however, the WT seedlings did not show marked difference in *PIN* expression levels from the untreated seedlings (Figure S3B). Data obtained from the root tips of Col (or *mthsc70-1a*) plants harboring *PIN:PIN-GFP* confirmed the above results. After treatment with 1 mM H_2_O_2_, the Col/*PIN1-*, *2-*, *3-* or *7-GFP* seedlings exhibited a marked decrease in PIN protein abundance in the root tips compared to the untreated seedlings (Figure 7A,B); however, *mthsc70-1a*/*PIN1-*, *2-*, *3-* or *7-GFP* seedlings did not show an obvious difference in PIN protein abundance in the root tips from the untreated seedlings (Figure S4A,B). After treatment with 4 mM ASC or 300 nM GSH, the *mthsc70-1a*/*PIN1-*, *2-*, *3-* or *7-GFP* seedlings showed an increase in PIN protein abundance in the root tips compared to the untreated seedlings (Figure 7C,D); however, the Col/*PIN1-*, *2-*, *3-* or *7-GFP* seedlings did not show marked difference in PIN protein abundance in the root tips from the untreated seedlings (Figure S4C,D). The above results indicated that an imbalance towards a redox state might inhibit the expression of PIN genes and decrease the levels of PIN proteins in seedlings. Then, H_2_O_2_ or GSH were applied to Col or *mthsc70-1a* seedlings harboring *DR5:GFP* to clarify their effects on auxin response. After the Col/*DR5:GFP* seedlings were treated with 1 mM H_2_O_2_, the GFP fluorescence intensities in the root tips were not an obvious difference from those before treatment (Figure S5). After the *mthsc70-1a*/*DR5*:*GFP* seedlings were treated with 300 nM GSH, the GFP fluorescence intensities in the root tips were also not obvious difference from those before treatment (Figure S5), which indicated that exogenous redox reagents had no evident effect on local auxin abundances in root tips.

### 3.4. Introduction of ROS Scavenging Enzymes Rescues Polar Auxin Transport in mtHSC70-1 Mutant Roots

**Figure 8:**
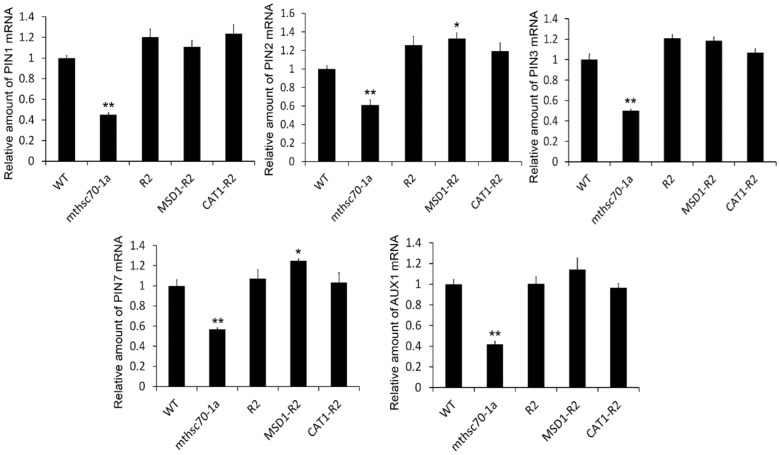
Introduction of the *mtHSC70-1*, *MSD1* or *CAT1* gene into *mthsc70-1a* rescued the expressions of auxin transport carrier genes in roots. Transcripts of *PIN1*, *PIN2*, *PIN3*, *PIN7* and *AUX1* genes in roots of 10 days old WT, *mthsc70-1a*, R2, MSD1-R2 and CAT1-R2 seedlings were analyzed by Q-PCR using gene-specific primers (Table S1). The data are shown as the means ± SD of three biological replicates. The asterisks indicate significant differences from the WT (*t*-test, *, *p <* 0.05; **, *p <* 0.01). WT, wild-type; *mthsc70-1a*, a *mtHSC70-1* mutant line; R2, a *mthsc70-1a*/*mtHSC70-1:mtHSC70-1* line; MSD1-R2, a *mthsc70-1a*/*mtHSC70-1:MSD1* line; CAT1-R2, a *mthsc70-1a*/*mtHSC70-1:CAT1* line.

**Figure 9:**
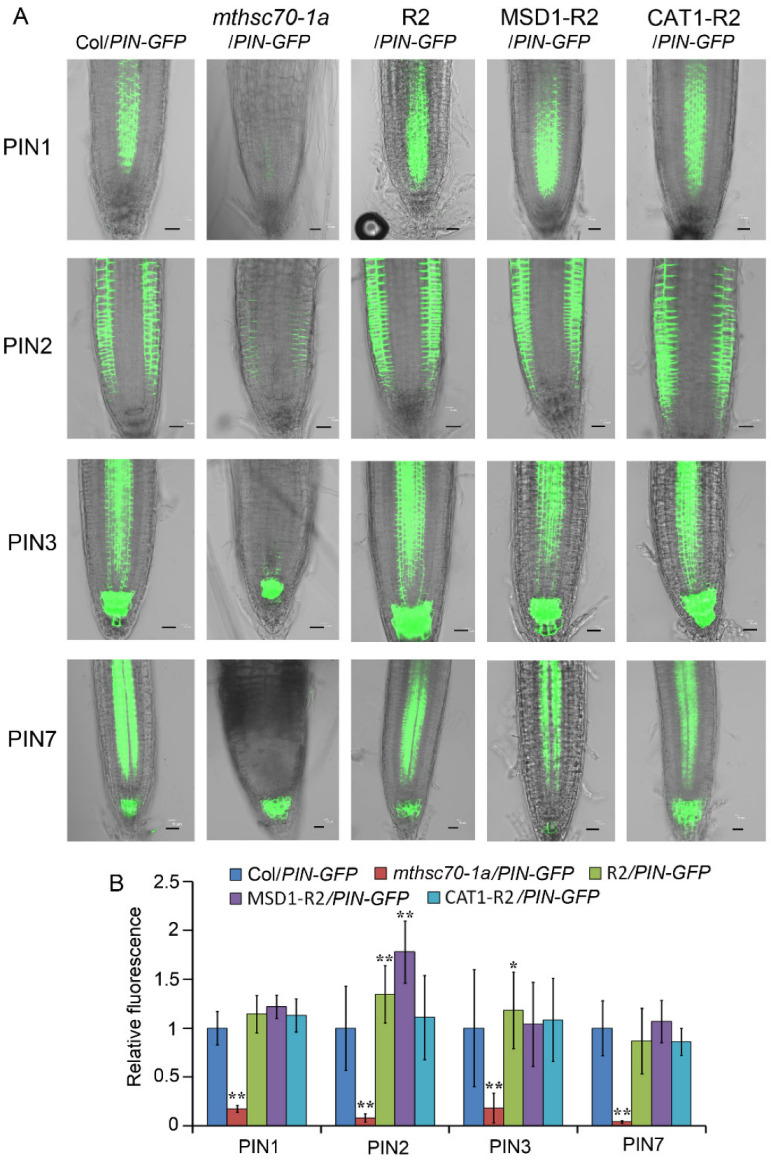
Introduction of the *mtHSC70-1*, *MSD1* or *CAT1* gene into *mthsc70-1a*/*PIN-GFP* plants rescued the levels of PIN proteins in root tips. (**A**) The roots of 5 days old seedlings with different genotypes were observed using a LSCM; representative images are shown. Scale bar = 20 µm. (**B**) The GFP fluorescence intensities were visualized with ImageJ 1.42q software (National Institutes of Health, Bethesda, MD, USA). The asterisks indicate significant differences from the Col/*PIN-GFP* plants (*t*-test, *, *p <* 0.05; **, *p <* 0.01). Col/*PIN-GFP*, Col/*PIN1-*, *2-*, *3- or 7-GFP* plants; *mthsc70-1a*/*PIN-GFP*, *mthsc70-1a*/*PIN1-*, *2-*, *3- or 7-GFP* plants; R2/*PIN-GFP*, R2/*PIN1-*, *2-*, *3- or 7-GFP* plants; MSD1-R2/*PIN-GFP*, MSD1-R2/*PIN1-*, *2-*, *3- or 7-GFP* plants; CAT1-R2/*PIN-GFP*, CAT1-R2/*PIN1-*, *2-*, *3- or 7-GFP* plants.

**Figure 10:**
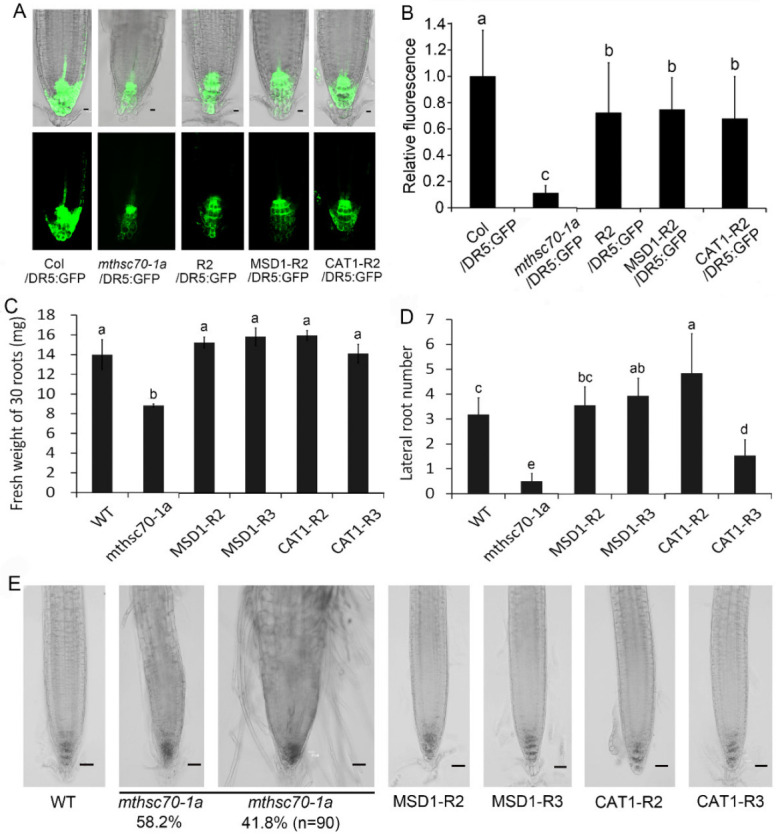
Introduction of the *mtHSC70-1*, *MSD1* or *CAT1* gene rescued the auxin response and root growth and development. (**A**) Root tips of 5 days old transgenic seedlings harboring *DR5:GFP* were observed using a LSCM; representative images are shown. Scale bar = 10 µm. (**B**) The GFP fluorescence intensities were visualized with ImageJ 1.42q software (National Institutes of Health, Bethesda, MD, USA). Thirty seedlings from each genotype were analyzed. The different letters indicate significant differences (ANOVA, Tukey’s HSD test, alpha *=* 0.05). (**C**,**D**) Comparison of fresh root weight (**C**) and lateral root number (**D**) among WT, *mthsc70-1a*, MSD1-R2, MSD1-R3, CAT1-R2 and CAT1-R3 seedlings. The different letters indicate significant differences (ANOVA, Tukey’s HSD test, alpha *=* 0.05). (**E**) Representative images of root tips of 10 days old seedlings with different genotypes. Scale bar = 40 µm. The roots of the *mtHSC70-1* mutants were divided into two types according to their width (≤140 µm or >140 µm) in the transition position of the meristem zone and elongation zone. The percentages below the images represent the ratio of two types of roots. n, the total number of roots analyzed. Col/*DR5:GFP*, Col plants harboring *DR5:GFP*; *mthsc70-1a/DR5:GFP*, *mthsc70-1a* plants harboring *DR5:GFP*; R2/*DR5:GFP*, R2 plants harboring *DR5:GFP*; MSD1-R2/*DR5:GFP*, MSD1-R2 plants harboring *DR5:GFP*; CAT1-R2/*DR5:GFP*, CAT1-R2 plants harboring *DR5:GFP*; WT, wild-type; *mthsc70-1a*, a *mtHSC70-1* mutant line; MSD1-R2 and MSD1-R3, two *mthsc70-1a*/*mtHSC70-1:MSD1* lines; CAT1-R2 and CAT1-R3, two *mthsc70-1a*/*mtHSC70-1:CAT1* lines.

*mtHSC70-1* knockout plants display increased levels of ROS in root tips [47]. To understand the effect of ROS accumulations caused by *mtHSC70-1* knockout on PAT, several complementary *mthsc70-1a* lines with *mtHSC70-1*:*mtHSC70-1* (R2 line), *mtHSC70-1*:*Mn-SOD1* (MSD1-R2 and MSD1-R3 lines) or *mtHSC70-1*:*CAT1* (CAT1-R2 and CAT1-R3 lines) were used to analyze the expressions of *PIN* genes, auxin distributions and root growth. It was demonstrated that introducing *MSD1* or *CAT1* genes into *mthsc70-1a* plants decreases the level of superoxide and H_2_O_2_ in roots [47]. The results obtained by Q-PCR showed that *mtHSC70-1* knockout markedly downregulated the expression of the *PIN1*, *PIN2*, *PIN3*, *PIN7* and *AUX1* genes in roots, and the introduction of *mtHSC70-1*, *MSD1* or *CAT1* into *mthsc70-1a* rescued the expression levels of these genes (Figure 8). Furthermore, we obtained R2/*PIN1-*, *2-*, *3- or 7-GFP*, MSD1-R2/*PIN1-*, *2-*, *3- or 7-GFP* and CAT1-R2/*PIN1-*, *2-*, *3- or 7-GFP* lines through the crossing method and compared the PIN protein levels among different genotypic plants. The PIN levels in *mthsc70-1a*/*PIN1-*, *2-*, *3- or 7-GFP* root tips were substantially lower than those in Col/*PIN1-*,*2-*,*3- or 7-GFP* root tips; the PIN levels in R2/*PIN1-*, *2-*, *3- or 7-GFP*, MSD1-R2/*PIN1-*, *2-*, *3- or 7-GFP* and CAT1-R2/*PIN1-*, *2-*, *3- or 7-GFP* root tips reached levels equivalent to those in Col/*PIN1-*, *2-*, *3- or 7-GFP* root tips or were higher than those in Col/*PIN1-*, *2-*, *3- or 7-GFP* root tips (Figure 9). In addition, we obtained R2/*DR5*:*GFP*, MSD1-R2/*DR5*:*GFP* and CAT1-R2/*DR5*:*GFP* lines through the crossing method and compared the GFP fluorescence intensities among Col/*DR5*:*GFP*, *mthsc70-1a*/*DR5*:*GFP*, R2/*DR5*:*GFP*, MSD1-R2/*DR5*:*GFP* and CAT1-R2/*DR5*:*GFP* lines. The GFP fluorescence intensity in *mthsc70-1a*/*DR5*:*GFP* root tips was markedly weaker than that in Col/*DR5*:*GFP*; however, the GFP fluorescence intensities in the R2/*DR5*:*GFP*, MSD1-R2/*DR5*:*GFP* and CAT1-R2/*DR5*:*GFP* root tips were obviously stronger than those in *mthsc70-1a*/*DR5*:*GFP* and were slightly weaker than that in Col/*DR5*:*GFP* (Figure 10A,B). These data indicated that the decreases in PIN and local auxin abundance caused by *mtHSC70-1* mutation were likely due to a high oxidation status, suggesting that mitochondrial ROS homeostasis is very important for maintaining PAT in the cytosol. Finally, the phenotypic analysis showed that the introduction of the *MSD1* or *CAT1* gene into *mthsc70-1a* plants rescued root growth and development, which was reflected in fresh root weight, lateral root number, root width, cell arrangement and root hair formation (Figure 10C–E), suggesting that the defects of the *mtHSC70-1* mutant roots may be attributed to an imbalance towards an oxidative redox state.

## 4. Discussion

Mitochondria are the major sites of energy production and link redox metabolism to ATP synthesis through the respiratory electron transport chain, which rely on the oxidation of glycolysis product. Glucose plays an important role in energy metabolism as a substrate of glycolysis reaction. Data presented here indicate that *mtHSC70-1* mutation leads to root growth and development defects, and glucose supplementation restores some phenotypes of the *mtHSC70-1* mutant, such as root width, root cell arrangement and root hairs, but has no effect on root length, fresh weight and lateral root number, suggesting the role of glucose in organ architecture. In previous studies, we unexpectedly found an increase in the O_2_ consumption rate and ATP level in the *mtHSC70-1* mutant [47]. Analysis of respiratory pathways in the mutant indicates an inhibition of the cytochrome c oxidase (COX) pathway and the activation of the alternative respiratory (AOX) pathway [47]. The higher O_2_ consumption rate caused by the increased AOX pathway in the *mtHSC70-1* mutant can compensate for the lower efficiency of ATP generation when electrons are channels through AOX [47]. Thus, the root growth defects in the *mtHSC70-1* mutant may not be explained by the lack of energy, which might be the reason for the observed weak restoring effect of glucose on root growth phenotypes.

Mitochondria are also major sites of ROS production. In the process of electron transfer in the respiratory chain, a small number of electrons do not complete the whole transfer chain but leak and directly transfer to O_2_, generating ROS [54]. Complex I, Complex II and complex III are generally regarded as the main and direct sources of ROS [54,55,56,57,58,59]. It is reported that complex IV-deficient mutants can create electron leakage at the site of over-reduced complex III [60]. Yeast cells lacking Rcf1 (a structural subunit of yeast COX) and Arabidopsis mitochondria-localized *CYTOCHROME C OXIDASE DEFICIENT1* (*COD1*) mutants display decreased COX/complex IV activity and increased ROS production [60,61]. Our previous study indicates that *mtHSC70-1* mutants exhibit reduced COX/complex IV activity and the accumulation of mitochondrial ROS, which might be responsible for shortened stems and roots and decreased rosette sizes [47]. Data presented here indicate that the introduction of ROS scavenging enzymes into *mtHSC70-1* mutant rescues the growth and development of the roots, suggesting an inhibition role of high levels of ROS in plant growth and development.

It is reported that mtHSC70-1 regulates embryo development by auxin pathway in *A. thaliana* [49], and *mtHSC70-1* knockout inhibits the growth of *A. thaliana* seedlings [47]. Whether mtHSC70-1 regulates vegetative growth through auxin is unclear. Data presented here demonstrate that mtHSC70-1 facilitates root growth and development through regulating PAT. Li et al. [49] reported that the loss of mtHSC70-1 function induces mitochondrial retrograde signaling, which results in abnormal auxin gradients and defective embryo development. However, the mitochondrial retrograde regulatory factor is still unknown. Our previous work demonstrated that the loss of mtHSC70-1 function leads to the accumulation of mitochondrial ROS [47]. Data presented here demonstrate that the removal of excess mitochondrial ROS in the mutant could rescue defective PAT. How mitochondrial ROS affect auxin transport and distribution in the cytosol and whether they play a role as the main mitochondrial retrograde regulator remain to be studied. It is reported that isolated mitochondria can release H_2_O_2_ from the matrix to the extra-mitochondrial space [8]. However, the rate at which this occurs physiologically in intact cells remains unclear.

It is reported that the expression of *CAT1* and *MSD1* is rapidly induced after treatment with H_2_O_2_, and catalase and SOD activity increase obviously after treatments with oxidative stressors in Arabidopsis [62,63,64]. Ślesak et al. [65] reported that three *PAD* downregulated transgenic lines display an increased H_2_O_2_ level and higher activities of MSD1 and CAT in the leaves and shoots compared to the wild-type plants. Previously, we found that the expression levels of *MSD1* and *CAT1* genes in the *mthsc70-1a* seedlings are obviously high than those in WT, and ROS levels are also markedly high in the mutant [47], indicating that endogenous *MSD1* and *CAT1* are induced by the ROS, and endogenous MSD1 and CAT1 are not enough to eliminate the excess ROS in the mutant. Further, treatments with antioxidants ASC and GSH do not restore the growth of *mthsc70-1a* and *mthsc70-1b* roots [47]. Data presented here indicate that the application of exogenous antioxidants has no obvious effect on the local auxin abundances in root tips. This may be because the applications of exogenous antioxidants lack spatiotemporal specificity. Then, we investigated the effects of introducing ROS scavenging enzymes into the *mthsc70-1a* mutant on the PIN and auxin abundances and root growth. There are three catalase genes *(CAT1*, *CAT2* and *CAT3)* and one manganese superoxide dismutase gene (*MnSOD* or *MSD1*) in the Arabidopsis genome [66,67]. Subcellular localization studies have suggested that MSD1 is localized in mitochondria [68,69]. Bioinformatics analysis shows that CAT1 is mainly localized in the peroxisome, mitochondria and cytosol (http://bar.utoronto.ca/cell_efp/cgi-bin/cell_efp.cgi?primaryGene=At1g20630) (accessed on 3 December 2017). To ensure the effective removal of excess ROS produced by mtHSC70-1 deficiency, we introduced the mitochondria-targeted *MSD1* or *CAT1* gene, which are driven by the *mtHSC70-1* promoter, into the *mthsc70-1a* mutant. The introduced *MSD1* or *CAT1* gene is expressed to some extent in several representative transgenic lines, and total *MSD1* or *CAT1* levels in the transgenic lines recover to a level similar to that in WT [47], which implies that ROS levels in these transgenic lines are decreased by the introduced *MSD1* or *CAT1*. Data presented here show that the introduction of *MSD1* or *CAT1* into *mtHSC70-1* mutants rescued the defects in *PIN* expression, auxin distribution and root growth and development. These results indicate that spatially and temporally distributions of ROS are essential for PAT and provide direct evidence for the role of ROS homeostasis in maintaining PAT.

## 5. Conclusions

**Figure 11:**
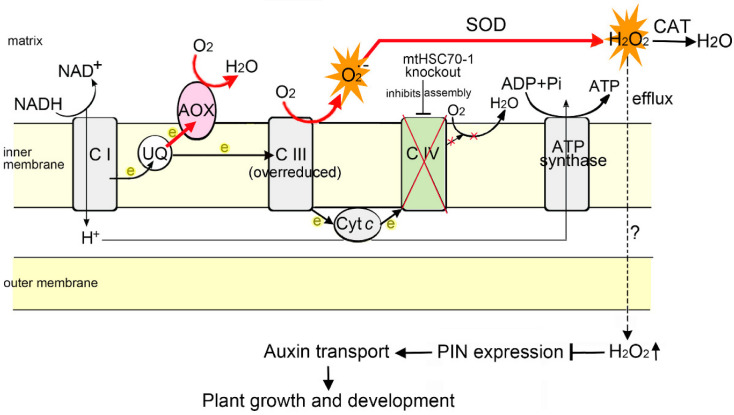
A working model for the function of mtHSC70-1 in the regulation of Arabidopsis growth and development. mtHSC70-1, as a molecular chaperone, assists in complex IV/cytochrome *c* oxidase (COX) assembly. The loss of mtHSC70-1 function inhibits the assembly/activity of COX, which results in the malfunction of the cytochrome respiratory pathway, which leads to the excessive production of ROS at the site of over-reduced complex III, and the activation of the alternative respiratory pathway. The high levels of ROS in *mtHSC70-1* mutants interfere with polar auxin transport, subsequently impacting the growth and development of plants. Inhibited and increased pathways are indicated with black lines and red lines, respectively. The bars show blockage effects. Black dashed line indicates an unproven route. Complex IV and electron transfer disruption is shown with red crosses. CI, CIII and CIV, respiratory chain complexes I, III and IV; AOX, alternative oxidase; UQ, ubiquinone; Cyt *c*, cytochrome *c*; SOD, superoxide dismutase; CAT, catalases.

Our previous results [47,48] and the data presented here support a working model in which mtHSC70-1 acts to assist respiratory chain complex IV assembly/activity and regulate ROS homeostasis, which maintains normal PAT and regulates plant growth and development (Figure 11).

## Data Availability

Data is contained within the article and Supplementary Material.

## Supplementary Materials

The following supporting information can be downloaded at: https://www.mdpi.com/article/10.3390/antiox11102035/s1. Figure S1. Comparison of root tip structure among wild type, *mthsc70-1a* and a complementary *mthsc70-1a* line. Figure S2. The effects of NAA treatment on primary root elongation in WT and *mtHSC70-1* mutant seedlings. Figure S3. Effects of exogenous redox reagents on the expression of *PIN genes* in *mtHSC70-1* mutant and WT seedlings by Q-PCR. Figure S4. Effects of exogenous redox reagents on PIN protein levels in *mthsc70-1a*/*PIN-GFP* and Col/*PIN-GFP* root tips. Figure S5. Effects of exogenous redox reagents on the auxin response. Table S1. List of primers used in this study. Table S2. Genotypes of the transgenic lines used in this study.
