# Supplementary material for: Mitochondrial HSC70-1 Regulates Polar Auxin Transport through ROS Homeostasis in Arabidopsis Roots
Source: Antioxidants (Basel). 2022 Oct 15;11(10):2035. doi: 10.3390/antiox11102035 (PMC9598091; doi:10.3390/antiox11102035)
Supplement: Supplementary file 1 [file antioxidants-11-02035-s001.zip › antioxidants-1940761-supplementary.pdf]

Supplementary data

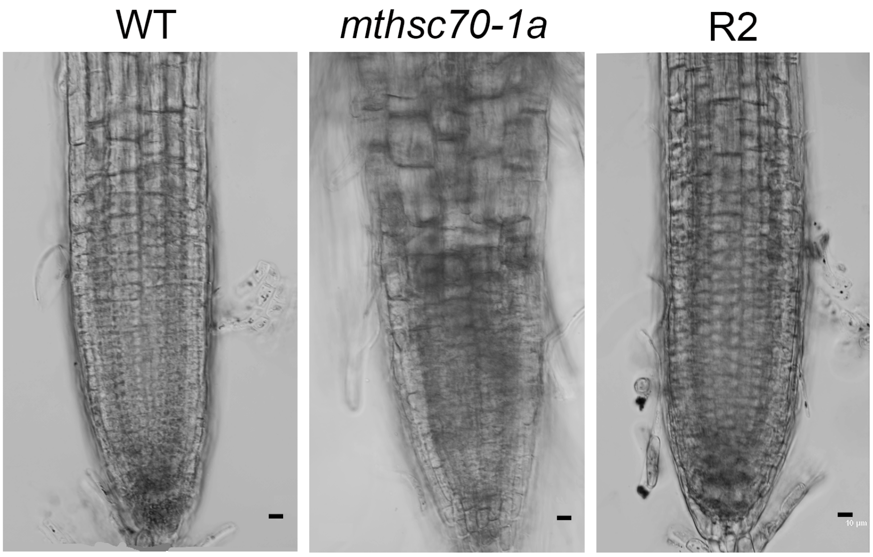

Figure S1. Comparison of root tip structure among wild type, *mthsc70-1a* and a complementary *mthsc70-1a* line. The roots of seven days old seedlings grown on 0.5 × MS medium supplemented with 1% sucrose were observed using a LSCM. Representative images are shown. Scale bar = 10 μm.

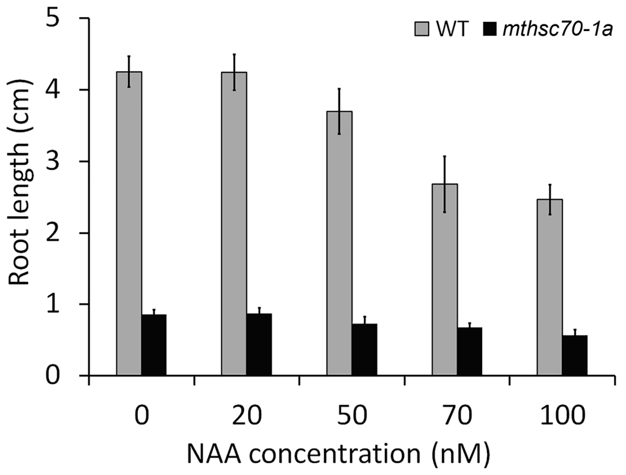

Figure S2. The effects of NAA treatment on primary root elongation in WT and *mtHSC70-1* mutant seedlings. Comparison of primary root length between 10-day-old WT and *mthsc70-1a* seedlings grown on 0.5 × MS medium supplemented with different concentrations of NAA. The data are the means ± SD of 30 seedlings. The experiments were repeated three times with similar results. WT, wild-type; *mthsc70-1a*, a *mtHSC70-1* mutant line.

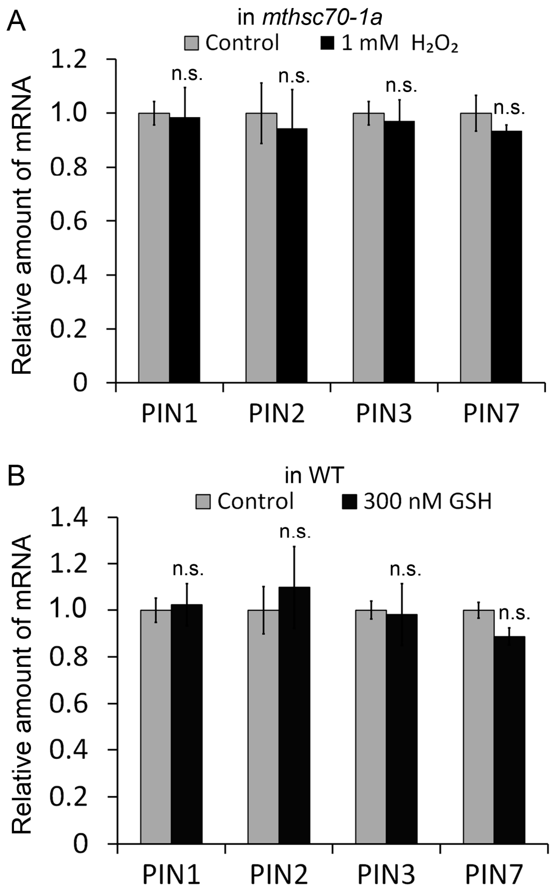

Figure S3. Effects of exogenous redox reagents on the expression of *PIN genes* in *mtHSC70-1* mutant and WT seedlings by Q-PCR. (A) The *mthsc70-1a* seedlings were grown on 0.5 × MS medium with or without 1 mM H_2O_2 for 10 d. (B) The WT seedlings were grown on 0.5 × MS medium with or without 300 nM GSH for 10 d. The data are the means ± SD of three biological replicates. n.s., no significant differences from control group; WT, wild-type; *mthsc70-1a,* a *mtHSC70-1* mutant line.

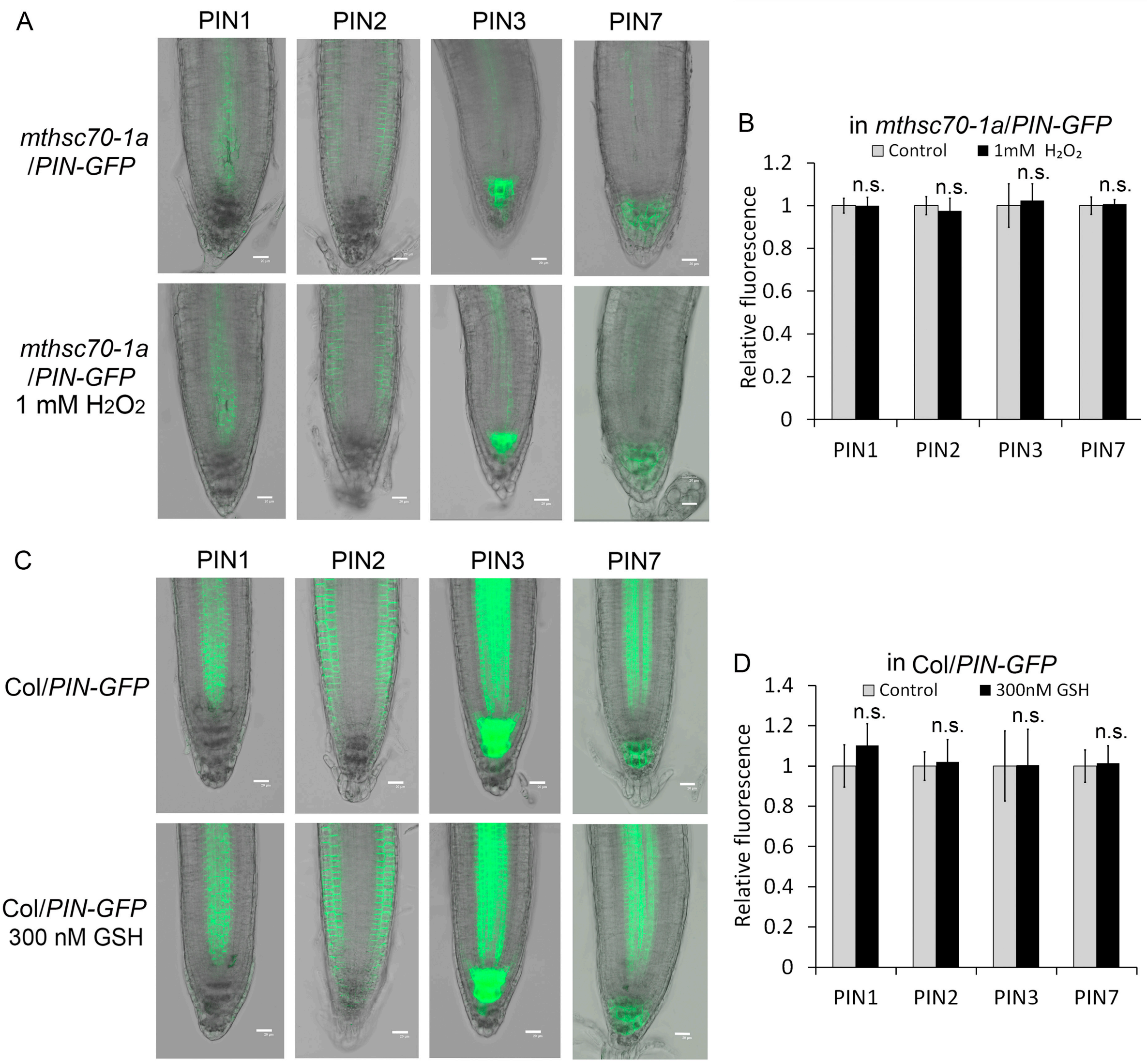

Figure S4. Effects of exogenous redox reagents on PIN protein levels in *mthsc70-1a/PIN-GFP* and Col/*PIN-GFP* root tips. (A, B) Root tips of five days old *mthsc70-1a/PIN-GFP* seedlings grown on 0.5 × MS medium with or without 1 mM H_2O_2 were observed using a LSCM (A); the GFP fluorescence intensities were visualized with ImageJ 1.42q software (National Institutes of Health, Bethesda, MD, USA) (B). (C, D) Root tips of five days old Col/*PIN-GFP* seedlings grown on 0.5 × MS medium with or without 300 nM GSH were observed using a LSCM (C); the GFP fluorescence intensities were visualized with ImageJ 1.42q software (D). Thirty seedlings from each genotype and treatment were analyzed; representative images are shown. Scale bar = 20 μm. n.s., no significant differences from control group; Col/*PIN-GFP*, Col/*PIN1-, 2-, 3- or 7-GFP* plants; *mthsc70-1a/PIN-GFP*, *mthsc70-1a/PIN1-, 2-, 3- or 7-GFP* plants.

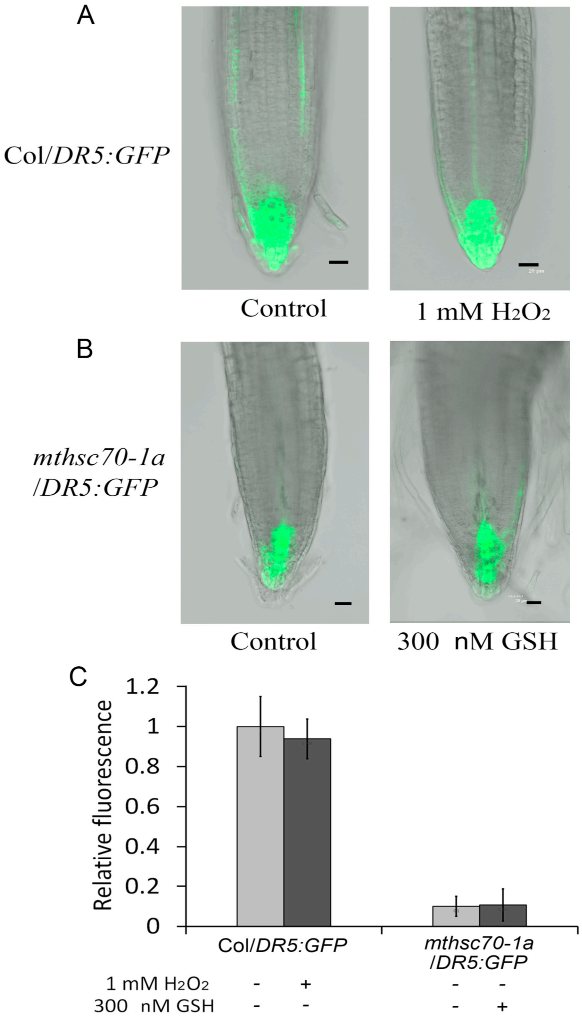

Figure S5. Effects of exogenous redox reagents on the auxin response. (A) Root tips of five days old Col/*DR5:GFP* seedlings grown on 0.5 × MS medium with or without 1 mM H_2O_2 were observed using a LSCM. (B) Root tips of five days old *mthsc70-1a*/*DR5:GFP* seedlings grown on 0.5 × MS medium with or without 300 nM GSH were observed using a LSCM. (C) The GFP fluorescence intensities were visualized with ImageJ 1.42q software (National Institutes of Health, Bethesda, MD, USA). Thirty seedlings from each genotype and treatment were analyzed; representative images are shown. Scale bar = 20 µm. Col/*DR5:GFP*, Col plants harboring *DR5:GFP*; *mthsc70-1a*/*DR5:GFP*, *mthsc70-1a* plants harboring *DR5:GFP*.

Table S1. List of primers used in this study

Locus number	Gene	Primer sequences (5´ to 3´)
At1g73590	*PIN1*	F: CTGGTCCCTCATTTCCTTCAAGTG
		R: TGAACAACCCAAGACTGAACATAGC
At5g57090	*PIN2*	F: TCCTCCATATCGCCATCGTTCAG
		R: AGCATTCCGAATATAACCGCAGTG
At1g70940	*PIN3*	F: ATGGCGGTTAGGTTCCTTACGG
		R: GCAAACACAAAGGGCACAATTCC
At1g23080	*PIN7*	F: AGTGTGATGACTCGGCTGATATTG
		R: ATCCCACCTGAAAGCAACAAGAG
At2g38120	*AUX1*	F: TGCCTCCGCTCGTCAG AATG
		R: CACCGAACCCAAATCCGACTATAAG
At2g37620	*ACTIN1*	F: GGTAACATTGTGCTCAGTGGTGG
		R: AACGACCTTAATCTTCATGCTGC
SALK_081383	*mthsc70-la*	L: AACACTGCAATGACACCTTCC
		R: AGGATTGAAATTACCCATCGG
SALK_081385	*mthsc70-1b*	L: AACACTGCAATGACACCTTCC
		R: AGGATTGAAATTACCCATCGG
	LBb1.3	ATTTTGCCGATTTCGGAAC

Table S2. Genotypes of the transgenic lines used in this study

Name of transgenic plant	Genetic background		Transgenic construct						
	Col	*mthsc70-1a*	*mtHSC70-1:mtHSC70-1*	*mtHSC70-1:MSD1*	*mtHSC70-1:CAT1*	*DR5:GUS*	*DR5:GFP*	*PINx:PINx-GFP*	*AUX1:AUX1-YFP*
R2, R3		Y	Y						
Col/*DR5:GUS*	Y					Y			
mthsc70-1a/DR5:GUS		Y				Y			
Col/*DR5:GFP*	Y						Y		
mthsc70-1a/DR5:GFP		Y					Y		
Col/*PIN-GFP*	Y							Y	
mthsc70-1a/PIN-GFP		Y						Y	
Col/*AUX1-YFP*	Y								Y
mthsc70-1a/AUX1-YFP		Y							Y
MSD1-R2, MSD1-R3		Y		Y					
CAT1-R2, CAT1-R3		Y			Y				
R2/*PIN-GFP*		Y	Y					Y	
MSD1-R2/*PIN-GFP*		Y		Y				Y	
CAT1-R2/*PIN-GFP*		Y			Y			Y	
R2/*DR5:GFP*		Y	Y				Y		
MSD1-R2/*DR5:GFP*		Y		Y			Y		
CAT1-R2/*DR5:GFP*		Y			Y		Y		

Note: Col, *Arabidopsis thaliana* (Columbia-0); *mthsc70-1a*, T-DNA insertion mtHSC70-1 knockout plants; *PINx*, *PIN1*, *PIN2*, *PIN3* or *PIN7*; Y, yes.
